# LabKey Server NAb: A tool for analyzing, visualizing and sharing results from neutralizing antibody assays

**DOI:** 10.1186/1471-2172-12-33

**Published:** 2011-05-27

**Authors:** Britt Piehler, Elizabeth K Nelson, Josh Eckels, Sarah Ramsay, Karl Lum, Blake Wood, Kelli M Greene, Hongmei Gao, Michael S Seaman, David C Montefiori, Mark Igra

**Affiliations:** 1 LabKey Software, Seattle, Washington 98102, USA; 2 Statistical Center for HIV/AIDS Research & Prevention (SCHARP), Fred Hutchinson Cancer Research Center, Seattle, Washington 98109, USA; 3 Department of Surgery, Duke University Medical Center, Durham, NC 27710, USA; 4 Division of Viral Pathogenesis, Beth Israel Deaconess Medical Center, Harvard Medical School, Boston, Massachusetts 02115, USA

## Abstract

**Background:**

Multiple types of assays allow sensitive detection of virus-specific neutralizing antibodies. For example, the extent of antibody neutralization of HIV-1, SIV and SHIV can be measured in the TZM-bl cell line through the degree of luciferase reporter gene expression after infection. In the past, neutralization curves and titers for this standard assay have been calculated using an Excel macro. Updating all instances of such a macro with new techniques can be unwieldy and introduce non-uniformity across multi-lab teams. Using Excel also poses challenges in centrally storing, sharing and associating raw data files and results.

**Results:**

We present LabKey Server's NAb tool for organizing, analyzing and securely sharing data, files and results for neutralizing antibody (NAb) assays, including the luciferase-based TZM-bl NAb assay. The customizable tool supports high-throughput experiments and includes a graphical plate template designer, allowing researchers to quickly adapt calculations to new plate layouts. The tool calculates the percent neutralization for each serum dilution based on luminescence measurements, fits a range of neutralization curves to titration results and uses these curves to estimate the neutralizing antibody titers for benchmark dilutions. Results, curve visualizations and raw data files are stored in a database and shared through a secure, web-based interface. NAb results can be integrated with other data sources based on sample identifiers. It is simple to make results public after publication by updating folder security settings.

**Conclusions:**

Standardized tools for analyzing, archiving and sharing assay results can improve the reproducibility, comparability and reliability of results obtained across many labs. LabKey Server and its NAb tool are freely available as open source software at http://www.labkey.com under the Apache 2.0 license. Many members of the HIV research community can also access the LabKey Server NAb tool without installing the software by using the Atlas Science Portal (https://atlas.scharp.org). Atlas is an installation of LabKey Server.

## Background

Standardized techniques for measuring neutralizing antibody (NAb) response to infectious agents are important tools for studies that address pathogenesis and vaccine development. Without proper standardization, variability in techniques can obscure differences in the quality and quantity of antibody responses. Luciferase-based reporter gene assays have been developed to assess the activity of a range of neutralizing antibodies [1-5]. For example, the TZM-bl NAb assay for HIV-1, SIV and SHIV provides a standard method for measuring neutralization and identifying promising antibodies for the development of HIV vaccines and immunotherapeutics [1]. This assay has been widely adopted by collaborating labs throughout the HIV research community [6-9] because it is sensitive, quantitative, high-throughput, reproducible, accurate, swift, and validated for good clinical lab practices (GCLP)[1,7].

**Figure 1:**
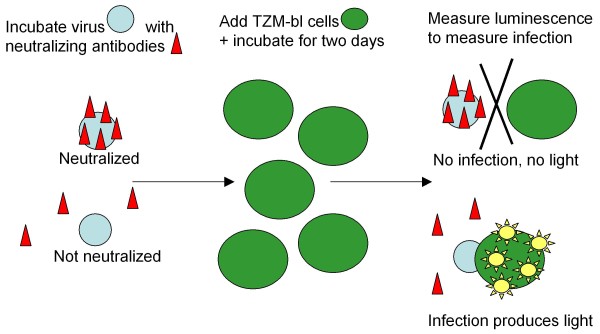
**Diagram of neutralization assay steps**. A virus is incubated for an hour with neutralizing antibodies, then TZM-bl cells are added and the mixture is incubated for an additional two days. The TZM-bl cells have been genetically modified with Tat-inducible luciferease, so they will luminesce when infected. The extent of infection is measured by observing the luminescence of these cells. High levels of luminescence indicate high levels of infection, so antibodies have not had success in neutralizing the virus, leaving it free to infect cells.

Figure 1 diagrams how the TZM-bl cell line is used to measure the success of antibodies in neutralizing HIV in the TZM-bl assay. The TZM-bl (JC53-bl) cell line has been genetically altered to express HIV entry receptors (CD4, CXCR4 and CCR5) and contain Tat-inducible luciferase (Luc) and β-galactosidase reporter genes. In the TZM-bl assay, virus and antibodies are incubated together at a range of concentrations, then combined with TZM-bl cells vulnerable to infection. After a further two days of incubation, measurement of relative luminescence units (RLU) compared to controls indicates the success of neutralization. Greater success in neutralization produces lower infection and thus lower luminescence. The concentration of antibodies necessary to noticeably reduce infection (*e.g.*, to achieve 50% neutralization) is used as a measure of the efficacy of the antibody. Measurement of luminescence usually takes place on one or many multi-well plates. Plates include virus control and cell control groups, plus replicates for each dilution. The luminometer outputs raw data in a standardized format directly into an Excel spreadsheet.

In the past, an Excel macro has been used to analyze these spreadsheets and calculate neutralization curves. The macro must be installed manually on every computer where researchers wish to use it. If updates to calculation procedures or plate layout patterns are needed, they must be applied on every computer where the macro is used. Result and source files must be painstakingly named to allow association of raw data and completed analyses. Files can quickly proliferate on multiple, distributed computers unless careful centralization, archiving and sharing procedures are in place and dutifully followed.

We present a standardized software tool and data management system for neutralizing antibody assays that can enhance the reproducibility, reliability, comparability and retention of results for assays such as TZM-bl. The tool makes it easy to apply standardized calculations through a web-based interface; centrally archive both data and results; and securely share this information between collaborating labs and beyond. Updates to the tool can be made once to the central server and appear uniformly to all users. A graphical plate designer makes it easy to design customized plate templates for new, high-throughput experiments and make them available to all users of the central server.

## Implementation

### Architecture

LabKey Server [10,11] is a web application implemented in Java. It runs on the Apache Tomcat web server and stores its data in a relational database, either PostgreSQL or Microsoft SQL Server. LabKey Server has been tested on computers running Microsoft Windows and most Unix variants, including Linux, Macintosh OSX and Solaris.

LabKey Server's NAb tool is packaged in a Java-based module that encapsulates user interface elements and calculation logic for designing, processing and displaying structured assays of various kinds.

Details of LabKey Server's architecture and assay module have been covered elsewhere [10]. LabKey Server v11.1, available in April 2011, is the 20^th ^official, public release of the platform since 2005.

## Results

### Overview of NAb tool

Users are prompted to provide the NAb tool with a raw instrument file produced by their luminometer, plus relevant metadata about the batch, run and samples that have been processed. Required metadata include characteristics necessary for calculations (*e.g.*, initial dilution, dilution factor and desired curve fit method). Optional metadata include characteristics of the experiment that can be used for tracking purposes (*e.g.*, experiment performer, incubation time and virus name). Both raw data and metadata are analyzed automatically by the system to estimate the titers at which specified levels of neutralization are seen for each sample/virus combination. Results can be integrated with other data types (such as specimen information and clinical data for study participants). The NAb tool displays neutralization curves, titers and source data in a secure, interactive, web-based interface.

### Options for leveraging the NAb tool

The LabKey Server NAb tool can be accessed in two ways:

• By logging into to the Atlas Science Portal. This option is available to participating member consortia of the Global HIV Enterprise [10,12,13].

• By installing and configuring a LabKey Server instance of your own. This option allows you to administer and customize your own, private LabKey Server for your lab or consortium.

Detailed information for these options is available in the "Availability and Requirements" section of this paper.

### Tutorials and documentation

Full documentation and tutorials for setting up, configuring and using LabKey Server and its NAb assay are available at http://www.labkey.org. This documentation is updated regularly to match the currently released version of LabKey Server. The LabKey Server NAb assay tutorial [14] provides a detailed walk-through and sample data.

### Assay plate options

**Figure 2:**
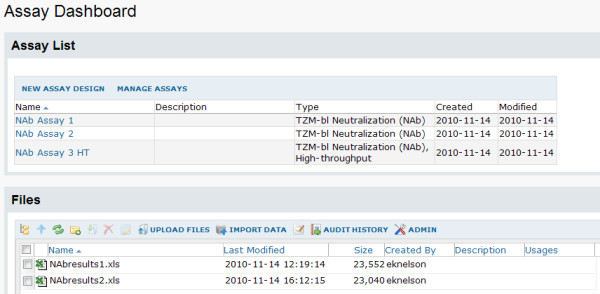
**Assay dashboard**. The assay dashboard provides a central location for managing different assays and their associated runs. This dashboard has been customized to include a list of all assay designs available in the current folder. It also displays a file management interface for uploading files and importing file data to appropriate assay designs.

The tool supports both low-throughput (single-plate) and high-throughput (multi-plate) NAb assays performed in either a 96-well or 384-well plate format. At present, a low-throughput assay typically utilizes a 96-well plate, usually prepared with five specimens in eight dilutions of two replicates each, as shown in Figure 2. A high-throughput assay typically uses 384-well plates, allowing for more samples per run. A high-throughput run may consist of up to eight plates. High-throughput samples are diluted across plates; in contrast, low-throughput samples are diluted within a single plate. Plate layout may be customized for both low- and high-throughput assays.

### Calculation of results

The NAb tool calculates the percent neutralization for each dilution or concentration after subtraction of background activity and fits a curve to the neutralization profile. It then uses this curve to calculate neutralizing antibody titers for chosen benchmarks, area-under-the-curve (AUC) and error estimates. Five-parameter, four-parameter, polynomial, and point-based methods are all currently used to calculate curve fits. For each method of curve fit, the tool calculates up to three inhibitory dilutions or inhibitory concentrations, according to the cutoff percentages chosen by the user. For example, the user might ask the tool to calculate IC20, IC50 and IC80, the concentrations at which the antibody inhibits 20%, 50% and 80% of infections. The tool also uses the fitted curve to calculate the AUC, the positive-area-under-the-curve (PostiveAUC) and curve fit error estimates. AUC is the total area under the curve, with negative regions counting against positive regions. PositiveAUC includes only the areas under the curve that are above the x-axis.

In addition to calculating curve-based neutralization titers, the tool also calculates "point-based" titers according to the method of Reed and Muench [15]. This is done by linearly interpolating between the two replicates on either side of the target neutralization percentage.

Formal comparisons between five-parameter, four-parameter, polynomial and point-based methods for calculating titers are not currently available. In general, five-parameter, four-parameter and polynomial methods provide more averaging than point-based methods, so they generate smoother trend lines. Common techniques for calculating five-parameter, four-parameter and polynomial curve fits are described elsewhere [16,17].

As part of ongoing development of the tool, new calculation techniques (such as a five-parameter curve fit) have been added to the tool and quickly deployed to all users. When desirable, new calculations (such as area-above-the-curve calculations) have automatically been added to results views for existing data. This meant that new insights into analysis techniques could quickly and transparently be applied to existing data, alongside old techniques. Automated and manual testing are performed before every public release of LabKey Server to ensure that analyses performed with new versions of the software produce results consistent with previous releases.

### Usage scenario for the LabKey Server NAb tool

**Figure 3:**
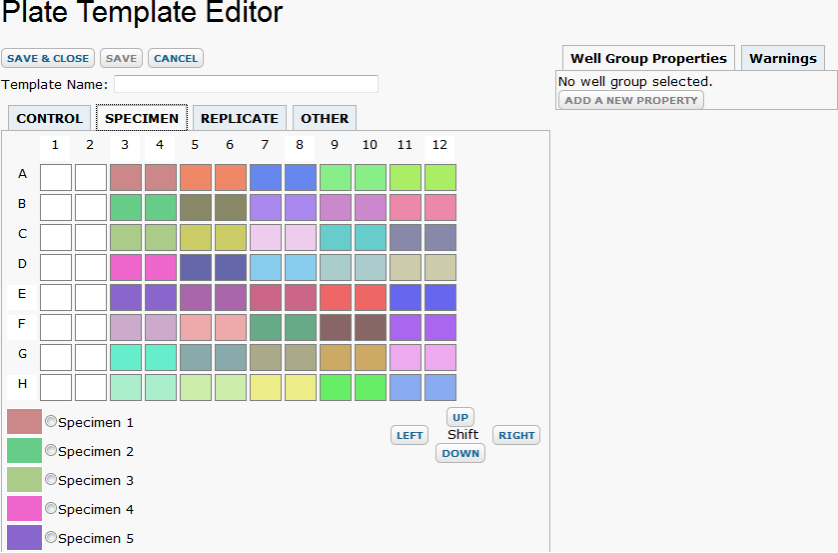
**Plate template editor**. The LabKey Server plate template editor allows you to indicate which wells contain particular specimens, replicates and controls. Processing of sample data depends on the layout of each plate, as indicated through a plate template. The plate template editor's graphical interface allows you to select a specimen and then drag across the plate template editor to "paint" with the chosen specimen. This makes it easy to edit large plate templates. A 96-well template is displayed here; a 384-well template is also available.

**Figure 4:**
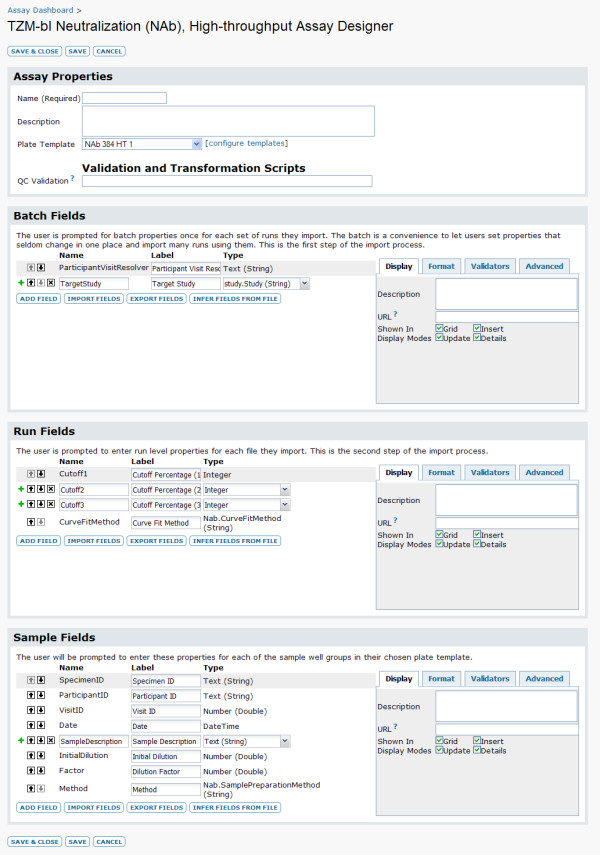
**Assay designer**. The LabKey Server assay designer [10] provides a pre-prepared assay design for both low- and high-throughput TZM-bl NAb assays. Both the plate template and the fields for this design can be customized. This figure shows the default design for high-throughput NAb experiments.

**Figure 5:**
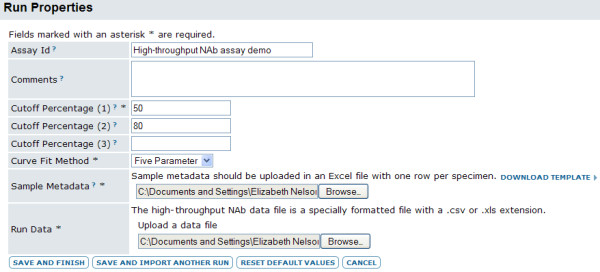
**NAb assay import screen**. This image shows how users indicate metadata and cut-off percentages for neutralization titers for a high-throughput NAb assay.

**Figure 6:**
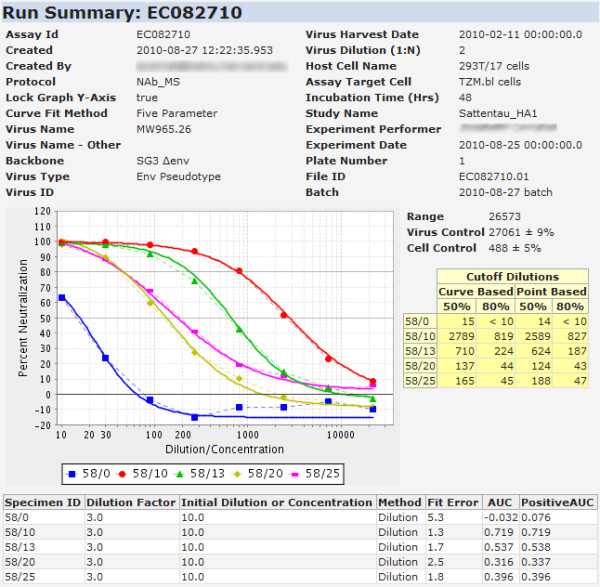
**LabKey Server NAb results view**. The calculated results of a NAb run are displayed in the "Run Summary." This example shows a low-throughput NAb run that has been shared publicly on the Atlas server as part of a completed CAVD study [19].

To use the NAb tool, a scientist typically follows these steps:

1. **Set up an assay folder**. After setting up a LabKey Server, an administrator creates an assay-type folder with appropriate permissions for user access. The home page for the folder shown in Figure 2 includes the Assay Dashboard (the starting place for uploading and processing NAb data) and the File web part (the general-purpose tool for uploading and importing files and data to the server).

2. **Configure a plate template**. A user next creates a plate template to match the design of the assay, or reuses an existing template. The template maps the contents of each well, including specimen controls and replicates. Figure 3 shows the plate template editor.

3. **Create an assay design**. With a plate template in hand, the user next creates an assay design that includes both this plate template and appropriate fields, such as "Sample ID" and "Initial Dilution," as shown in Figure 4. This design is used as the framework for uploading many individual assay runs.

4. **Upload files and import data**. After creating an assay design, a user can begin uploading files to the repository and simultaneously importing data from those files into the database. A part of this process, the assay design guides the collection of appropriate metadata for the assay run, as shown in Figure 5. This information is used to determine data processing and to map samples to plate locations. Information collected typically indicates whether the sample is being diluted or concentrated; the initial dilution or concentration of each sample; the dilution factor; 1-3 cut-off percentages for calculation of inhibitory concentrations or dilutions; a unique sample identifier; and the default curve fit method.

5. **View results**. Curve fits and related results are calculated automatically for each assay run during the import process. Figure 6 shows a results view for a low-throughput NAb assay. Calculated results are displayed towards the top of the page, while raw plate data are provided at the bottom. The results page shows calculations for inhibitory concentration (or dilutions), areas under the curves and the standard deviations of controls, both viral and cell. A drop-down menu allows users to choose which curve fit mechanism is used to calculate displayed results.

### Data integration and quality control

**Figure 7:**
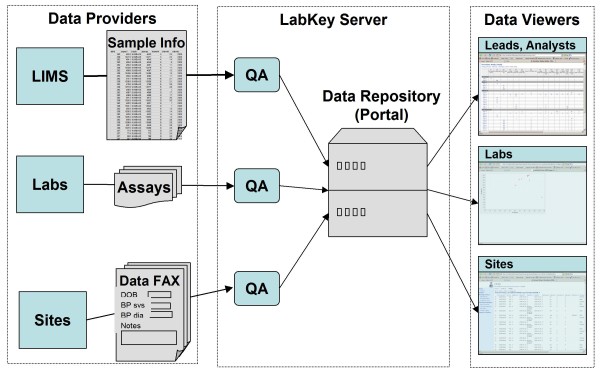
**LabKey Server data flows**. This figure shows how data flows into a LabKey Server in many forms (*e.g.*, Excel, text and DataFax case report forms) from many sources (*e.g.*, labs, clinics and repositories). Once within the system, data can be accessed by collaborators (*e.g.*, labs, principal investigators and statisticians) in desired formats (*e.g.*, data grids, custom R views or charts) through a web-based portal.

LabKey Server allows researchers to integrate information from NAb assays with other study information through sample, participant and visit identifiers [10]. The platform also provides mechanisms for performing quality control before sharing NAb results widely and integrating these results with other data sources. Figure 7 shows a schematic overview of how data can flow into a LabKey Server from diverse sources, undergo quality control and become available to a range of data customers through a web-based portal.

Typically, imported NAb data includes sample identifiers (sample IDs). These are used to look up the participant IDs and visit IDs associated with the samples during assay run import. Data are initially stored in a private folder, along with sample IDs, participant IDs and visit IDs. Quality control checks are then performed to identify inconsistencies in the data and users can see the errors immediately onscreen.

After the data have been reviewed privately for accuracy, they are typically then transferred to a shared integration folder called a study. A study allows groups of researchers to use participant and visit IDs to integrate data of many different types, such as specimen information, clinical data and other assay results for a study participant. Just like NAb data, all of these data types have first been reviewed for accuracy before they are transferred to a study folder for sharing and integration.

### Additional features of LabKey Server

LabKey Server includes a range of additional built-in features that can be helpful to users of the NAb tool. These include a fine-grained security model, auditing, a robust API and tools for data integration. A full review of LabKey Server's features as of v10.3 is available elsewhere [10].

The platform's folder- and role-based security model allows a single LabKey Server to support multiple groups privately managing NAb analyses in protected projects and folders. Data can be shared based on the role and group memberships of each user. Furthermore, finished data can be released publicly by simply lowering the permissions requirements for the particular subfolder that contains the data.

LabKey Server's assay infrastructure supports auditing and can be used to achieve GCLP. For example, imported runs can be re-imported to the system as new runs for error correction, but existing runs cannot be edited.

NAb data stored on a LabKey Server can be accessed through LabKey's rich API. This API supports a variety of programming languages, including JavaScript and Perl, which makes it easy for labs to write custom tools that work with data (including NAb data) stored on a LabKey Server.

### Current Usage

LabKey Server's NAb tool has been adopted by multiple consortia within the Global HIV Enterprise [10,12,13] for uploading, processing and sharing results of the TZM-bl NAb assay. These labs use the tool as part of the Atlas Science Portal [18], which is itself an installation of LabKey Server. The NAb tool has been used successfully by 14 labs across 5 organizations within the Enterprise: the Collaboration for AIDS Vaccine Discovery (CAVD), the Center for HIV Vaccine Immunology (CHAVI), the Vaccine Immunology Statistical Center (VISC), the HIV Vaccine Trials Network (HVTN) and the U.S. Military HIV Research Program. As of January 2011, these labs have used the NAb assay tool to upload and store over 40,000 NAb assay runs. As of May 2010, Atlas supported approximately 2,800 user accounts originating in roughly 350 organizations and 36 countries [10].

Post-publication sharing of NAb assay analyses is a particularly notable aspect of how the LabKey Server NAb tool is being used. Multiple labs within the CAVD consortium have made their NAb data and analyses public on the Atlas portal [18]. This portal provides an interactive interface that provides sortable and filterable grid views of results. Figure 6 shows an example of a NAb assay results view available publicly on Atlas [19]. Results from other CAVD studies can be explored using the CAVD study design browser [20].

## Discussion

### Overview of advantages

Robust software tools can make it easier for assay techniques to be standardized and transferred uniformly to multiple labs, as long as they can be updated swiftly and consistently to reflect new techniques. Server-based tools like the LabKey Server NAb tool can be delivered as software-as-a-service (SaaS) and deployed and updated uniformly, unlike Excel-based macros. Advantages of the LabKey Server NAb tool extend beyond uniform deployment. The NAb tool also provides configurable support for evolving workflows and high-throughput techniques. Furthermore, it provides a secure, web-based platform for archiving, integrating and sharing data and files, features unavailable with Excel alone.

### Software-as-a-service

Updates to the LabKey Server NAb tool (such as the addition of a new curve fit algorithm) can be made efficiently and transparently on a central web server. Unlike a macro, updates do not need to be installed on a multiplicity of machines. This eliminates worries about which version of the macro was used to calculate results; instead, results are displayed centrally using the latest version of the tool and algorithms. At the same time, upgrading the LabKey Server NAb tool does not overwrite results calculated at the time of data import; these original results are retained in particular views. Optionally, new algorithms (such as a new calculation of "area under the neutralization curve") can be applied automatically to all archived data. This makes it swift and easy to augment old analyses with data from new techniques.

### Template customization

The flexibility of LabKey Server's NAb tool makes it easier to customize and refine than an Excel macro. LabKey Server's assay framework provides a graphical plate template designer that makes it easy to adjust the data formats understood by the NAb tool and share new templates with other users. Easy template customization is particularly helpful when using swiftly-evolving, high-throughput techniques.

### A central data repository

Data and results that live in a central, secure repository (such as a LabKey Server) are not lost when a technician, student or scientist leaves the research program, or when a single computer is lost. LabKey Server's granular permissions framework makes it easy for collaborating researchers to pool data in a secure environment, or to share subsets of data publicly after publication.

### Usage for other assays

The LabKey Server NAb tool described here can be used for other 96-well and 384-well plate-based assays beyond the TZM-bl NAb assay. These might include assays that utilize optical density, luminescence, fluorescence, radio activity and other quantitative measures as end points. Moreover, the tool is not limited to assays for NAbs. Using an existing, refined tool like LabKey Server can help researchers standardize on a common platform for many assays across many studies and labs. Furthermore, researchers can avoid writing new, custom macros for similar assays.

There are several constraints to applying this tool to other assays. To use the existing tool for other assays, experiments must conform to specific plate layouts that can be described with the tool. Furthermore, analyses of such assays must conform to the patterns supported by the tool.

As mentioned previously, code modifications to the tool itself can also be made to support additional, custom needs of such assays. The degree of difficulty of these modifications depends on their scope.

### Future Directions

As high-throughput NAb assays evolve, the LabKey team will continue to customize the NAb tool to new needs. There is interest in extending the tool to support multi-virus experiments. Today, the tool assumes that each plate contains a single virus control. Future work would generalize the tool further to analyze plates that contain multiple virus controls.

Beyond the NAb tool, the LabKey team will continue to develop new, built-in assays for the platform. LabKey Server's assay framework and robust API has also been used by third-party developers to produce new assay modules in languages such as JavaScript [10]. LabKey Software provides ongoing support and development of the LabKey Server platform.

## Conclusions

The LabKey Server NAb tool provides a standardized tool for calculating, storing and sharing data from low- and high-throughput NAb experiments. These experiments are essential for identifying subtle differences in antibody success in neutralization of HIV-1, SIV and SHIV and ultimately for developing novel vaccines and treatments for AIDs. The LabKey Server NAb tool provides a wide range of advantages over the Excel macro that has been used in the past for analyzing TZM-bl NAb assay data. These advantages include standardized deployment across labs, swift customizability and a web-based interface for organizing, integrating, archiving and sharing data and files.

Scalable, robust tools for analyzing and managing experimental results in a standardized manner will only become more valuable as distributed teams of researchers use high-throughput techniques to produce ever-increasing volumes of data. The open source license for LabKey Server and its NAb tool allow other researchers to freely leverage, customize and improve this tool.

## Availability and Requirements

### LabKey Server Open Source and Compiled Binaries

The LabKey Server open source software is freely available for download at http://www.labkey.org under the terms of the Apache License 2.0 [21]. This site also provides documentation, tutorials and demos for users and developers, plus instructions for developers who wish to contribute code to the project through the LabKey Subversion repository.

Compiled binaries for Windows, Unix, Linux or Macintosh installation are available for free through LabKey Software at http://www.labkey.com. A graphical installer is available for computers running Windows XP or later. It includes the LabKey Server web application; the Apache Tomcat web server, v5.5.29; the Java Runtime Environment, v1.6.0-22; the PostgreSQL database server, v8.3.7; and additional third-party components.

• **Project name**: LabKey Server

• **Project home page**: http://www.labkey.org

• **Operating system(s)**: Platform independent

• **Programming languages**: Java, JavaScript, R, Perl, SAS, *etc*.

• **Other requirements, as of LabKey v11.1**: Apache Tomcat (5.5.29, 5.5.31 or 5.5.32); Java Runtime Environment 6; and either PostgreSQL (8.2, 8.3 or 9.0) or Microsoft SQL Server (2005, 2008 or 2008 R2). Check the project site for latest requirements of the most recent release.

• **License**: Apache License 2.0 [21]

### Access to the Atlas Science Portal

Access to Atlas is available to participating members of the research networks as part of the Global HIV Enterprise. To inquire about access, contact atlas@scharp.org. Published NAb results are available to the public without logon at https://atlas.scharp.org. At present, many NAb results can be found in completed CAVD studies, which are located on Atlas in the VISC folder.

## Abbreviations

**AIDS**: Acquired immune deficiency syndrome; **AUC**: Area under the curve; **CAVD**: the Collaboration for AIDS Vaccine Discovery; **CHAVI**: the Center for HIV Vaccine Immunology; **the Enterprise**: the Global HIV Enterprise; **HIV**: Human immunodeficiency virus; **HVTN**: the HIV Vaccine Trials Network; **ID**: Identifier; **NAb assay**: Neutralizing antibody assay; **NIAID**: National Institute of Allergy and Infectious Diseases; **PositiveAUC**: Positive area under the curve; **SaaS**: Software as a Service; **SCHARP**: the Statistical Center for HIV/AIDS Research & Prevention at the Fred Hutchinson Cancer Research Center; **VISC**: the Vaccine Immunology Statistical Center

## Authors' contributions

BP designed and implemented the LabKey Server NAb tool. EKN wrote this paper and provided documentation and testing. JE contributed development of the platform's assay architecture. SR provided end-user training and assistance in support of tool adoption. KL advanced analytics. BW triaged and prioritized user requests.

KMG and HG contributed to the development of the NAb assay protocol and provided feedback on the tool. MSS provided key feedback on tool usage scenarios. DCM and his lab provided data from the TZM-bl assay and provided ongoing feedback. MI contributed prototyping and requirements gathering. All authors reviewed and approved this paper.
